# A PET/CT radiomics model for predicting distant metastasis in early-stage non–small cell lung cancer patients treated with stereotactic body radiotherapy: a multicentric study

**DOI:** 10.1186/s13014-024-02402-z

**Published:** 2024-01-22

**Authors:** Lu Yu, Zhen Zhang, HeQing Yi, Jin Wang, Junyi Li, Xiaofeng Wang, Hui Bai, Hong Ge, Xiaoli Zheng, Jianjiao Ni, Haoran Qi, Yong Guan, Wengui Xu, Zhengfei Zhu, Ligang Xing, Andre Dekker, Leonard Wee, Alberto Traverso, Zhaoxiang Ye, Zhiyong Yuan

**Affiliations:** 1 Department of Radiation Oncology, Tianjin Medical University Cancer Institute and Hospital, National Clinical Research Center for Cancer, Tianjin, China; 2 Department of Radiology, Tianjin Medical University Cancer Institute and Hospital, National Clinical Research Center for Cancer, Tianjin, China; 3 Zhejiang Cancer Hospital, Hangzhou Institute of Medicine (HIM), Chinese Academy of Sciences, Hangzhou, Zhejiang 310022 China; 4 Department of Radiation Oncology (Maastro), GROW School for Oncology and Reproduction, Maastricht University Medical Centre, Maastricht, The Netherlands; 5 The Affiliated Cancer Hospital of Zhengzhou University, Zhengzhou, China; 6 Department of Radiation Oncology, Fudan University Shanghai Cancer Center, Shanghai, China; 7 Department of Radiation Oncology, Shandong Cancer Hospital and Institute, Shandong First Medical University, Shandong Academy of Medical Science, Jinan, Shandong China

**Keywords:** Radiomics, PET/CT, Non-small cell lung cancer, Stereotactic body radiotherapy, Distant metastasis

## Abstract

**Objectives:**

Stereotactic body radiotherapy **(**SBRT) is a treatment option for patients with early-stage non-small cell lung cancer (NSCLC) who are unfit for surgery. Some patients may experience distant metastasis. This study aimed to develop and validate a radiomics model for predicting distant metastasis in patients with early-stage NSCLC treated with SBRT.

**Methods:**

Patients at five institutions were enrolled in this study. Radiomics features were extracted based on the PET/CT images. After feature selection in the training set (from Tianjin), CT-based and PET-based radiomics signatures were built. Models based on CT and PET signatures were built and validated using external datasets (from Zhejiang, Zhengzhou, Shandong, and Shanghai). An integrated model that included CT and PET radiomic signatures was developed. The performance of the proposed model was evaluated in terms of its discrimination, calibration, and clinical utility. Multivariate logistic regression was used to calculate the probability of distant metastases. The cutoff value was obtained using the receiver operator characteristic curve (ROC), and the patients were divided into high- and low-risk groups. Kaplan-Meier analysis was used to evaluate the distant metastasis-free survival (DMFS) of different risk groups.

**Results:**

In total, 228 patients were enrolled. The median follow-up time was 31.4 (2.0-111.4) months. The model based on CT radiomics signatures had an area under the curve (AUC) of 0.819 in the training set (*n* = 139) and 0.786 in the external dataset (*n* = 89). The PET radiomics model had an AUC of 0.763 for the training set and 0.804 for the external dataset. The model combining CT and PET radiomics had an AUC of 0.835 for the training set and 0.819 for the external dataset. The combined model showed a moderate calibration and a positive net benefit. When the probability of distant metastasis was greater than 0.19, the patient was considered to be at high risk. The DMFS of patients with high- and low-risk was significantly stratified (*P* < 0.001).

**Conclusions:**

The proposed PET/CT radiomics model can be used to predict distant metastasis in patients with early-stage NSCLC treated with SBRT and provide a reference for clinical decision-making.

**Plain language summary:**

In this study, the model was established by combining CT and PET radiomics signatures in a moderate-quantity training cohort of early-stage NSCLC patients treated with SBRT and was successfully validated in independent cohorts. Physicians could use this easy-to-use model to assess the risk of distant metastasis after SBRT. Identifying subgroups of patients with different risk factors for distant metastasis is useful for guiding personalized treatment approaches.

**Supplementary Information:**

The online version contains supplementary material available at 10.1186/s13014-024-02402-z.

## Introduction

Non-small-cell lung cancer (NSCLC) is the most common pathological type of lung cancer worldwide. Approximately 20% of NSCLC patients are in localized stages of disease (stages I and II) [1, 2]. Stereotactic body radiation therapy (SBRT), which delivers localized high doses in a few fractions, has become the standard of care for medically inoperable patients and early-stage NSCLC patients who do not wish to undergo surgery. It is well tolerated and provides high rates of local control [3, 4]. Nevertheless, distant metastasis is common in patients with early-stage disease. In the RTOG 0236 clinical trial, the distant metastasis rate was 27% in 55 patients with early-stage NSCLC [5]. Distant metastasis is highly correlated with poor prognosis, and the median survival of patients with metastatic NSCLC is only 6 months [6]. For these patients, chemotherapy, tyrosine kinase inhibitor (TKI)-targeted therapy, or immunological therapy may help improve progression-free and overall survival [7]. Therefore, early prediction of distant metastasis is necessary.

Radiomics aims to reveal tumor environment heterogeneity by mining medical images using artificial intelligence methods [8], which show great potential for predicting cancer prognosis [9]. Computed tomography (CT) and Magnetic Resonance Imaging (MRI) are important tools for detecting, diagnosing, staging tumor lesions, and contributing to clinical decision-making, follow-up, and prediction of cancer prognosis [10–14]. Molecular imaging, particularly ^18^F-Fluorodeoxyglucose positron emission tomography/computed tomography (^18^F-FDG PET/CT), is valuable for the evaluation and prediction of response, and is superior to morphological assessment by CT or MRI [15, 16]. Models based on CT radiomics features showed moderate performance in predicting the prognosis of patients with early-stage NSCLC treated with SBRT [17–19]. However, a PEF/CT-based model that predicts the prognosis of early-stage NSCLC is still lacking. PET/CT radiomics has been reported to successfully predict local recurrence in 87 early-stage NSCLC patients treated with SBRT [20]. Therefore, it is promising to use PET/CT radiomics features to predict the prognosis of patients with early-stage NSCLC treated with SBRT.

The purpose of this study was to develop and validate a radiomics model that contains signatures from pretreatment PET/CT for individualized prediction of distant metastasis in early-stage NSCLC patients treated with SBRT.

## Materials and methods

### Patients

This was a TRIPOD type 3 study. The external test sets were from several different hospitals, whereas the training set was from only one hospital (not included in the test set). In total, 139 patients with early-stage NSCLC treated with SBRT from the Tianjin Medical University Cancer Institute and Hospital were retrospectively included in the training set. For external validation, 20 early-stage NSCLC patients treated with SBRT from Zhejiang Cancer Hospital, 27 from the Affiliated Tumor Hospital of Zhengzhou University, 19 from Fudan University Shanghai Cancer Center, and 23 from Shandong Cancer Hospital and Institute were enrolled in the study. Details of the start and end dates of patient recruitment at the five institutions are provided in Supplementary Table S1. All the patients were diagnosed with early-stage NSCLC by a multidisciplinary team (MDT) and were recommended for SBRT because of contraindications to surgery. Surgical contraindications can be divided into two categories. First, patients were unable to tolerate surgery due to internal medical complications, including inadequate cardiopulmonary function, coagulation disorders, immunodeficiency, poor Eastern Cooperative Oncology Group (ECOG) performance status, severe systemic diseases, etc. Second, the patient or his/her relatives refused surgery after surgical evaluation by the thoracic surgeon. The inclusion criteria were as follows: (A) following the eighth American Joint Committee on Cancer classification, maximum tumor diameter less than 7 cm and stage I-II NSCLC; (B) pretreatment PET/CT imaging performed; (C) pathological diagnosis was not mandatory if the patient was unable to undergo biopsy due to contraindications. The exclusion criteria were as follows: (A) pretreatment PET/CT was performed at other hospitals, (B) the quality of PET/CT images was poor, (C) incomplete clinical data, (D) prior to SBRT, and patients who received other anticancer treatments.

**Fig. 1:**
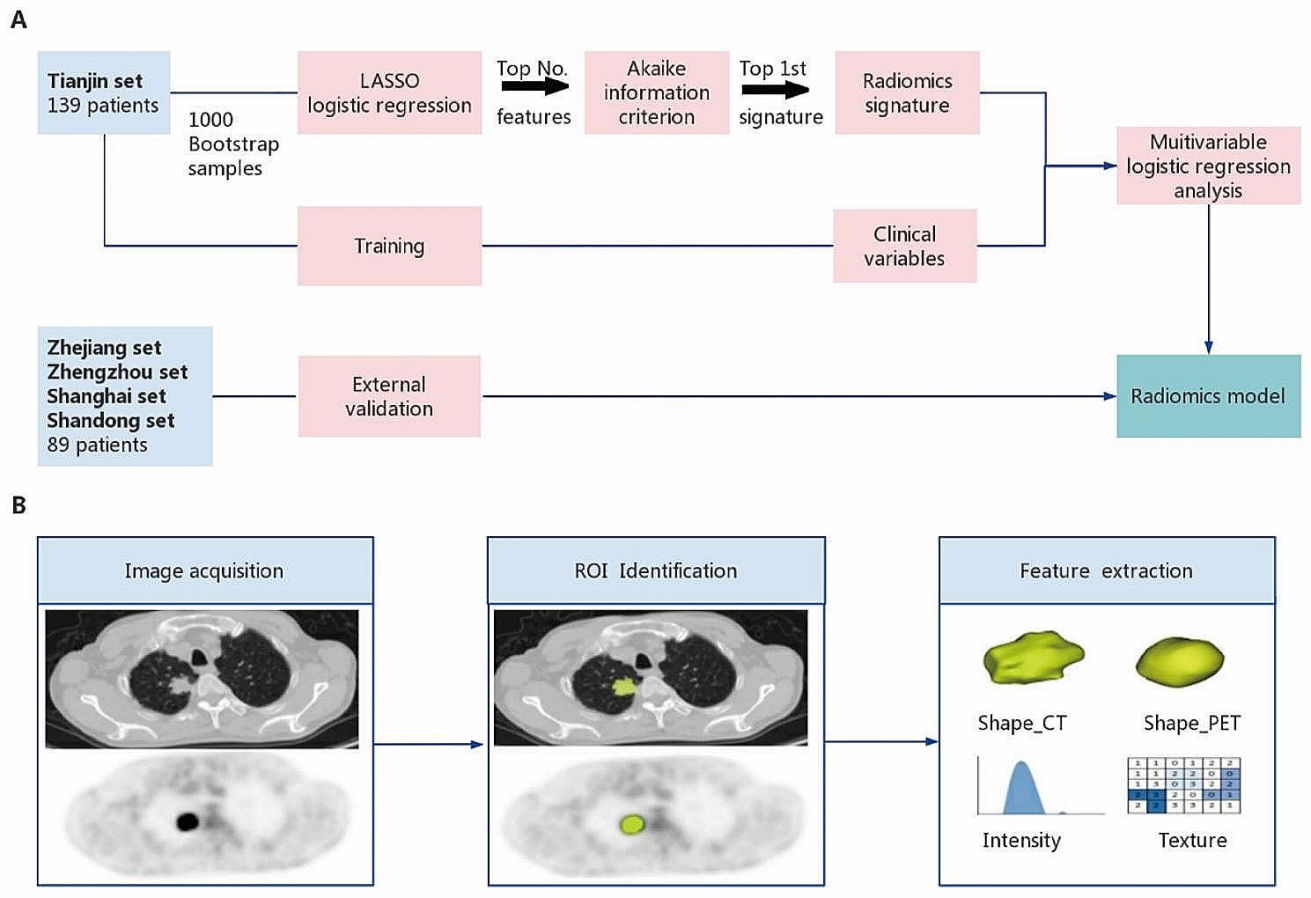
Study flowchart and radiomic workflow. (**A**) Study flowchart. (**B**) Radiomics workflow

The overall design of this study is illustrated in Fig. 1A and Figure S1, respectively. Distant metastasis was defined as (a) lesions in extrapulmonary organs such as the brain and liver or (b) multiple lung metastases according to RECIST v1.1 [21]. All processes in this study, including human participants, followed the 1964 Helsinki Declaration and its later amendments or comparable ethical standards.

Before SBRT was performed, demographic and clinical data were collected (Table 1), including sex, age, diagnosis date, smoking history, Eastern Cooperative Oncology Group (ECOG) performance status, Maximum Standardized Uptake Value (SUVmax), disease stage, lesion size measured on CT images, PET/CT diagnostic images, status at last follow-up, date and site of distant metastasis, and histology when available. Patients who had smoked less than 100 cigarettes before SBRT were defined as never smokers. Patients were subjected to regular follow-up following stereotactic body radiotherapy (SBRT), and the frequency of these examinations varied over time. In the initial 2 years post-SBRT, patients underwent follow-up every 3 months, which was subsequently extended to a 6-month interval from 2 to 5 years post-SBRT. Five years after SBRT, patients were annually followed up. At each follow-up, we conducted medical records review, physical examination, tumor marker testing, and chest CT scans.

**Table 1:** Baseline characteristics of the patients

Variables	Training set (*n* = 139)	External validation set (*n* = 89)	P value
Age(years)	72(46–89)	75(51–95)	0.577
Sex			0.090
Female	56(40.2)	26(29.2)	
Male	83(59.7)	63(70.7)	
Smoke			0.237
Yes	92(66.1)	52(58.4)	
No	47(33.8)	37(41.5)	
ECOG			0.020
0	72(51.7)	60(67.4)	
1	67(48.2)	29(32.5)	
T stage			0.183
T1	109(78.4)	74(83.1)	
T2	25(17.9)	15(16.8)	
T3	5(3.5)	0(0.0)	
Tumor size(cm)	2.2(1.0-6.1)	2.1(0.9–4.6)	0.124
Tumor location			0.293
Peripheral	132(94.9)	87(97.7)	
Central	7(5.0)	2(2.2)	
Histology			< 0.001
Adenocarcinoma	29(20.8)	42(47.1)	
Squamous cell carcinoma	22(15.8)	10(11.2)	
Undifferentiated NSCLC	8(5.8)	6(6.7)	
No pathology	80(57.5)	31(34.8)	
SUVmax	8.7(2.8–28.8)	6.2(2.9–21.8)	0.009
Radiation dose per fraction (Gy)	12(7–20)	10(5-12.5)	< 0.001
Total radiation dose (Gy)	60(48–60)	50(48–70)	< 0.001
BED_10_(Gy)	132(83.3–180)	105(75–119)	< 0.001
Distant metastasis			0.133
Yes	37(26.6)	16(17.9)	
No	102(73.3)	73(82.0)	

### PET/CT images acquirement, volumes of interest segmentation, feature extraction

The radiomics workflow is shown in Fig. 1B. PET/CT was performed in all patients within 45 days before the start of SBRT. Digital Imaging and Communications in Medicine (DICOM) data from pretreatment PET/CT were used for analysis. Images were segmented using the 3D Slicer software (version 4.13.0). Radiomics features were extracted using *Pyradiomics* package based on Python (version 3.7). In total, 103 CT radiomic features and 103 PET radiomic features of each lung lesion were extracted from the PET/CT images. Details regarding PET/CT acquisition and reconstruction, radiomics procedure, and radiomics features are described in Supplementary Material A1 and Tables S2 and S3.

### Feature selection, signature construction and performance assessment

Feature selection for the radiomics model was adapted from the feature pooling and signature pooling methods used by Compter et al. [22]. Briefly, the selection process was as follows:

(i) One thousand unique bootstrap samples (with replacement) were drawn from the training cohort. Within each bootstrap sample, we first minimized the number of strong pairwise normalized [Z-score, (original value-mean value)/(standard deviation)] feature correlations greater than 0.90 or less than − 0.90. A least absolute shrinkage (LASSO) loop with 20-times repeated 5-fold cross-validation embedded with a logistic regression (LR) supervised classifier was used to select the features. From each of the 1000 bootstraps, we ranked each individual feature according to how frequently it was retained by LASSO-LR.
(ii) Some of the most frequently appearing individual features were arbitrarily selected from the above table. From this small subset of selected features, we built a multivariable LR model for each of the aforementioned bootstrap samples with stepwise backward elimination using the Akaike information criterion (AIC) as a metric. From each of these 1000 bootstraps, we tabulated the number of times each combination of one or more features (i.e., potential signatures) was retained by the stepwise LR.
(iii) We arbitrarily selected the top most frequently appearing signature to build the final multivariable LR model. The coefficients of the final model were fitted using the original non-bootstrapped development cohort [23, 24].

Radiomics scores were calculated as linear combinations of the selected features weighted by the respective coefficients. Feature selection and radiomic score calculations were performed for the CT and PET scans, respectively. The Mann-Whitney U test was used to evaluate the differences in scores between the different patient subgroups.

### Model internal validation

We estimated over-optimism in the model development using the method recommended by the TRIPOD guidelines. For each of the 1000 abovementioned predefined bootstraps, we fitted the LR model coefficients on each bootstrap and then computed the Area under the curve (AUC) of the receiver operating characteristic curve (ROC) using the original non-bootstrapped development cohort. From these 1000 bootstraps, we computed the average AUC and 95% confidence interval (CI).

### Construction of the combined radiomics model

In the training set, PET score and CT score, of which P-values from univariable logistic regression analyses were less than 0.1, were subjected to consecutive multivariable analysis via the likelihood ratio test with entering selection. Based on the results of multivariable logistic analyses, a combined radiomics model was developed.

### Performance evaluation of the combined radiomics model and external validation

The discrimination performance of the radiomics model was quantified and visualized using AUC analysis. The radiomics scores for every lung lesion in the validation set were calculated using the formula constructed in the training set. To evaluate the goodness-of-fit of the model, calibration of the model was measured using a calibration curve accompanied by the Hosmer–Lemeshow test in both the training and external validation sets [25].

### Clinical validity of the combined radiomics model and risk grouping

To evaluate the improvement of performance by the radiomics signature, ROC analyses were performed in all cases to determine the contrast between the discriminant efficacy of the radiomics model and that of certain clinical parameters. Decision curve analysis (DCA) was conducted to identify the clinical usefulness of the combined radiomics model by measuring the net benefits at different threshold probabilities [26]. For clinical relevance, the dividing cutoff of distant metastasis probability was calculated using logistic regression, which was used to divide the patients into two risk groups. Survival curves for the risk groups are presented as Kaplan-Meier plots.

## Results

### Patient clinical characteristics

Table 1 shows the clinical information of patients in the training and external validation sets. In the training set, the median total radiation dose was 60 Gy (range, 48–60 Gy), the median fraction was 5 Gy (range, 3–8 Gy), and the median biologically equivalent dose (BED) was 132 Gy (range, 83.3–180 Gy). The median follow-up was 30.3 (range, 2.0-114.0) month. Distant metastasis occurred in 37 patients (26.6%) after a median follow-up of 21.9 (range, 2.0-83.9) months.

In the external validation set, the median total radiation dose was 50 Gy (range, 48–70 Gy), the median fraction was 5 Gy (range, 4–14 Gy), and the median BED was 105 Gy (range, 75–119 Gy). The median follow-up was 31.8 (range, 2.8–69.3) months. Distant metastasis occurred in 16 (17.9%) patients after a median follow-up of 18.7 (range, 2.8–62.8) months. The ECOG performance status, SUVmax, and histology were statistically significant between the training and external validation sets.

### Feature selection, radiomics signature selection and evaluation of model performance

For CT radiomics, the top 11 features were selected according to a frequency scatter plot (Table S4A, Figure S2A). Among the top 11 features, the most frequent signature was selected as the CT score model (Table S5A). For PET radiomics, the top 9 features were selected (Table S4B, Fig. S2B). The most frequent signature was the PET score model (Table S5B). Radiomics scores were calculated by screening the coefficients of features and intercepts, which are shown in Supplementary Material A2. Mann-Whitney U tests showed that patients with distant metastasis had higher CT scores than those without distant metastasis (Table S5).

The CT radiomics signature had moderate discrimination, with an AUC of 0.819 (95% CI, 0.745–0.892] in the training set and 0.786 (95% CI, 0.641–0.931) in the external validation set (Table 2). The PET radiomics signature had moderate discrimination, with an AUC of 0.763 (95% CI, 0.678–0.848) in the training set and 0.804 (95% CI 0.681–0.927) in the external validation set.

**Table 2:** Discrimination ability of radiomics signatures

Model	Training set (95%CI)	Over-optimistic correction (95%CI)	External validation set (95%CI)
CT score	0.819 (0.745–0.892)	0.804 (0.728–0.880)	0.786 (0.641–0.931)
PET score	0.763 (0.678–0.848)	0.735 (0.646–0.824)	0.804 (0.681–0.927)
CT score + PET score	0.835 (0.780–0.891)	0.828 (0.757–0.898)	0.819 (0.692–0.947)

### Construction of the combined radiomics model and assessment of performance

CT and PET scores were identified as independent predictors of distant metastasis after multivariable logistic regression analysis (Table 3). Therefore, the final prediction model for distant metastasis was established by combining CT and PET scores. The distant metastasis score of each patient was calculated according to the logistic regression formula: distant metastasis score = 0.474 + 0.891 × CT score + 0.570 × PET score. The probability of predicted distant metastasis was calculated using 1/ [1 + exp (distant metastasis score)].

**Table 3:** Potential predictors of distant metastasis in patients with early-stage NSCLC treated with SBRT

	Univariable logistic regression			Multivariable logistic regression	
Variables	OR (95%CI)	P		OR (95%CI)	P
CT score	2.526 (1.544–4.131)	< 0.001		2.435 (1.549–3.829)	< 0.001
PET score	1.929 (1.056–3.522)	0.033		1.769 (1.094–2.860)	0.020
Age(years)	0.979 (0.918–1.044)	0.523		-	-
Sex (female vs. male)	2.505 (0.779–8.054)	0.123		-	-
Smoke (no vs. yes)	0.852 (0.241–3.009)	0.804		-	-
ECOG (0 vs. 1)	0.749 (0.278–2.020)	0.569		-	-
Tumor size (cm)	0.896 (0.495–1.621)	0.716		-	-
Localization (central vs. peripheral)	2.037 (0.238–17.462)	0.516		-	-
Pathological diagnosis (yes vs. no)	1.608 (0.837–3.092)	0.154		-	-
SUVmax	1.010 (0.909–1.121)	0.855		-	-
BED_10_ (Gy)	1.001 (0.972–1.030)	0.961		-	-

SBRT, Stereotactic body radiotherapy; NSCLC, non-small cell lung cancer; ECOG, Eastern Cooperative Oncology Group; OR, odds ratio; CI, confidence interval; BED, biological equivalent dose, α/β = 10

**Fig. 2:**
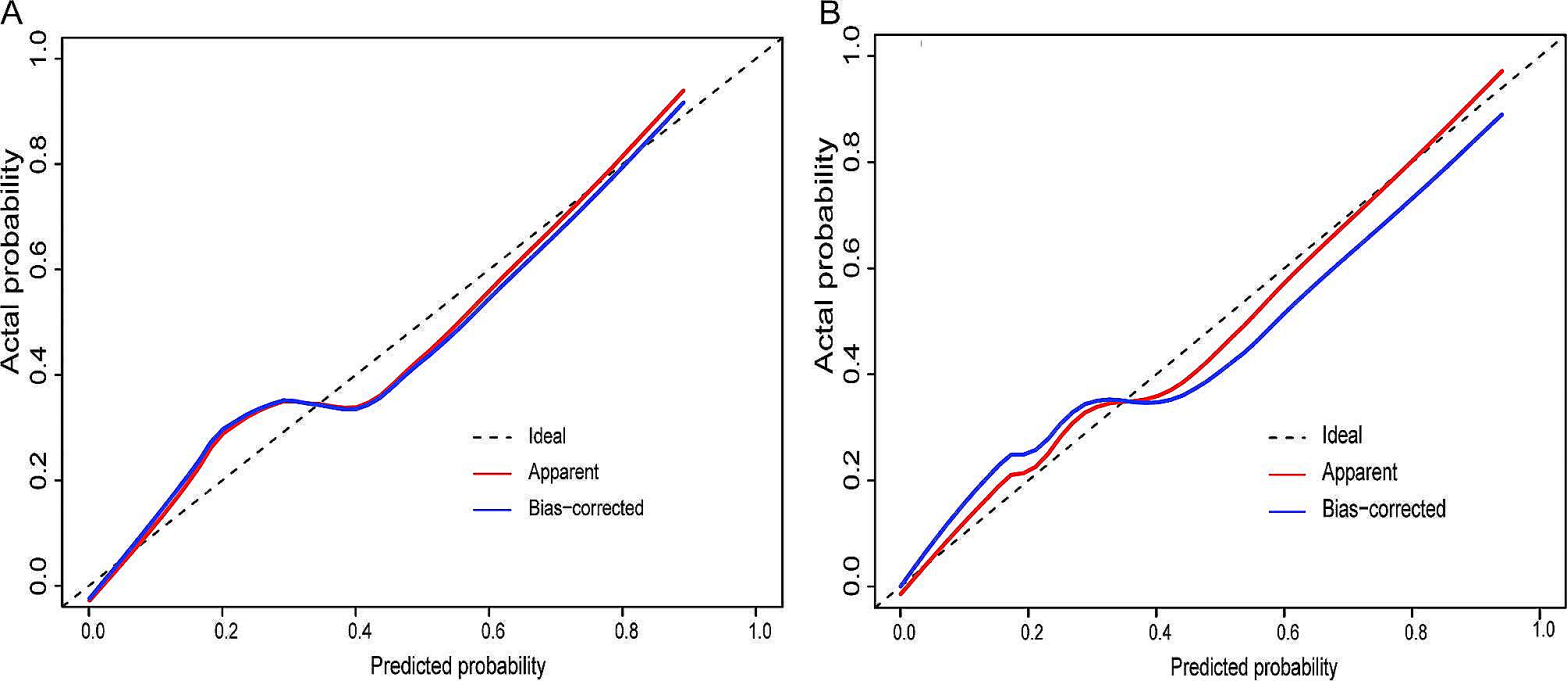
Calibration efficiency of the combined radiomics model. (**A**) In the training set. (**B**) In the external validation set

In the training set, the combined radiomics model had favorable calibration (Fig. 2A) and discrimination performance with an AUC of 0.835 [95% CI, 0.780–0.891]. The Hosmer–Lemeshow test was not statistically significant (*P* = 0.148).

### External validation of the combined radiomics model

In the external validation sets, the combined radiomics model had favorable calibration (Fig. 2B) and discrimination performance with an AUC of 0.819 [95% CI, 0.692–0.947] (Table 2). The Hosmer–Lemeshow test was not statistically significant (*P* = 0.219). The satisfactory results for the external validation set indicate that the model is universal.

### Clinical usefulness of the combined radiomics model

ROC curve analysis showed that the model combining PET signature with CT signature had better predictive performance than conventional clinical parameters, such as tumor size and SUVmax (Figure S3). In both the training and external validation sets, decision curve analyses showed that the use of the combined radiomics model to predict distant metastasis added more net benefits than the all-distant or non-distant metastasis regimens (Figure S4).

### Risk grouping

**Fig. 3:**
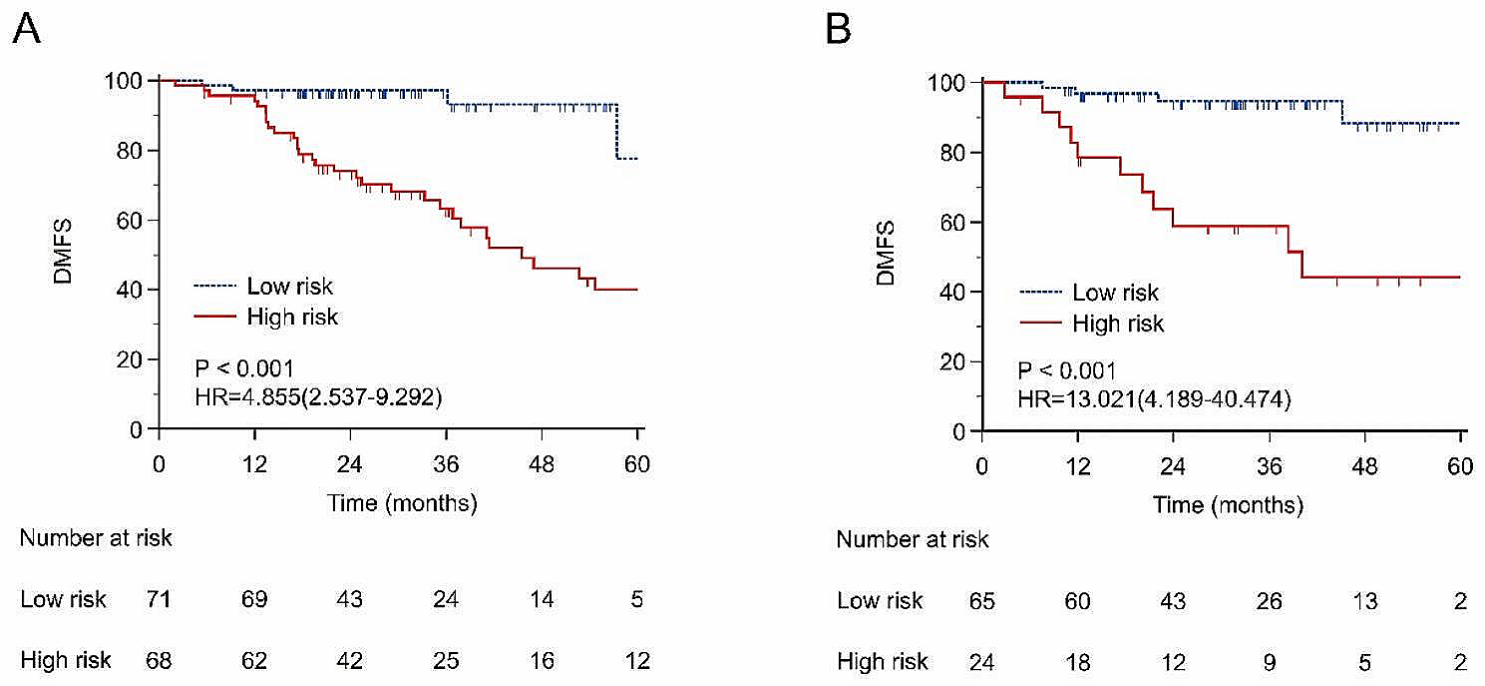
Kaplan-Meier analysis of distant metastasis free survival (DMFS) of the data set. (**A**) In the training set. (**B**) In the external validation set

In the training set, patients were divided into high- and low-risk groups according to the optimal cut-off value (0.19). The Kaplan-Meier plots suggested that the DMFS of patients in the training set (*P* < 0.001, Hazard ratio, HR = 4.855, 95% CI, 2.537–9.293) and the external validation set (*P* < 0.001, HR = 13.021, 95% CI, 4.189–40.474) were significantly stratified by risk grouping (Fig. 3).

## Discussion

In this research, the model was established through combining CT and PET radiomics in a moderate quantity training cohort of early-stage NSCLC patients treated with SBRT, and successfully validated in an independent cohort, thereby improving the generalizability and credibility. Physicians could utilize this easy-to-use model to assess the risk of distant metastasis after SBRT. Identifying subgroups of patients at different risk for distant metastasis is useful in guiding personalized treatment approaches. Patients at high risk of distant metastasis should be closely followed up or applied adjuvant therapy after SBRT. In addition, this model could provide information for patient stratification in the design of clinical study.

The AUC for the CT radiomics model was 0.819 for the training cohort and 0.786 for the external cohort. The AUC for the PET radiomics model was 0.763 for the training cohort and 0.804 for the external cohort. The discrepancy in AUC values between the two groups can be attributed to several factors, including variations in the data distribution, differences in image acquisition equipment, minor model overfitting, and the limited size of the external dataset. Despite these variations, the AUC values for the training and external datasets were remarkably similar, suggesting that the features learned by the model are likely to have good generalizability and are not specific to the training data. The high AUC values for both datasets indicate that the model successfully learned robust radiomics features from the images, which were associated with distant metastases and can be effectively generalized to new data. However, to thoroughly assess the model’s real-world clinical generalizability, more comprehensive validation on larger and more diverse datasets is needed. The coefficient of the CT score (0.891) was found to be greater than that of the PET score (0.570) when calculating the distant metastasis score. This suggested that the ability of CT to predict distant metastasis was superior to that of PET alone. This could be attributed to the higher resolution and clarity of lung tissue and lesions provided by CT images than by PET. Furthermore, CT imaging can offer a more detailed description of morphological features, particularly in the early stages of lung cancer. Additionally, CT imaging is capable of capturing tumor features such as density and texture, which are closely associated with tumor heterogeneity and aggressiveness, whereas PET primarily reflects tumor metabolic activity.

Gao et al. [27] established a nomogram for predicting distant metastasis within 1 year after SBRT by including 1280 patients from multiple centers. Despite the large number of enrolled patients, only clinical characteristics were considered. The AUC of predicting distant metastasis was 0.714 in the training set and 0.698 in the validation set, which was limited. Wu et al. [28] predicted distant metastases based on PET images by using 70 patients as the training set and 31 patients as the validation set. The consistency index of the PET radiomics model was 0.71. Li et al. [29] found that, among various radiomics methods based on machine learning algorithms, the model using the kernel-backed tensor machine (KSTM) algorithm had the highest predictive value (AUC = 0.84). However, the sample sizes of both studies were less than 150, and external validation was lacking. Whether these models can be applied to other institutions requires further exploration. This study possesses the advantage of utilizing radiomics features from both CT and PET scans, providing complementary information, unlike some other studies that rely solely on CT imaging. Moreover, the AUC of this study for predicting metastasis ranged from 0.819 to 0.835, demonstrating better discrimination performance than previous studies. Furthermore, the study proposed different criteria for high- and low-risk patients, distinguishing them from several other studies that developed models without specific risk stratification [29–31]. In future research, we will increase the sample size and incorporate methods such as deep learning. Additionally, integrating additional clinical parameters, genomic data, and radiomics features will enhance the accuracy of the model.

The quality of image resolution can vary tremendously from institution to institution [32]. To reduce the influence of the different PET/CT instruments of the five institutions, we used the Combat method to remove the batch effect, and the results showed that the performances of the models before and after Combat were similar [33]. This further supports the stability of our study. Local recurrence that may be influenced by factors related to the therapy itself will ultimately drive distant metastases [34]. Therefore, we excluded patients who had local recurrences from enrollment. Metachronous primary tumors can arise in up to 20% of patients with early-stage lung cancer. To exclude metachronous primary tumors, two radiologists with 10 years of experience determined the endpoint of distant metastasis. Intrapulmonary metastasis should be considered when two suspected malignant lesions show solid predominant lesions without spiculation or air bronchogram on CT [35].

Radiomics features are derived from medical images using specific algorithms, including intensity-based measures, first-order statistics, and heterogeneity and texture features [36]. Intensity measures and first-order statistics features were direct physical or functional measures from fully quantitative modalities and basic statistical measures characterizing the distribution of intensity values within an area, such as the mean of the image intensity values. The first-order statistic describes the distribution of the voxel intensities within the image region defined by the mask. The original _firstorder_RootMeanSquared (RMS) in the PET score is the square root of the squared mean of all intensity values. This is a measure of the image value size [37]. The smaller the RMS, the smaller the squared mean of all intensity values and the more homogeneous the intensity. The more homogeneous the composition of the tumor region, the less heterogeneous it is, and the less prone it is to distant metastasis. However, the definition of radiomics features is still vague, and many studies are still being conducted [38]. The values of texture features can reflect the heterogeneity of signal intensity within the lesion (e.g., GLCM, GLRLM, NGTDM, and GLSZM). Derived from the GLRLM in the CT score, original_glrlm_LowGrayLevelRunEmphasis reflects the connectivity of low gray-level regions within the image. The presence of more low-gray connected regions in an image is indicated by higher LGRE values. On CT images, regions of low density often indicate necrotic tissue or cystic degeneration. A low LGRE value suggested a shorter run length with a low gray level within the tumor tissue, indicating that necrotic and cystic areas may be more dispersed rather than continuous. This dispersion may reflect greater tumor heterogeneity, suggesting that the cell population within the tumor exhibits greater variability. It is important to note that greater tumor heterogeneity is associated with an increased risk of tumor invasion and metastasis [8, 39].

Distant metastasis can significantly affect patient survival. The 5-year overall survival rate of metastatic lung cancer is approximately 7% [40], it is necessary to predict distant metastasis in advance. The National Comprehensive Cancer Network (NCCN) guidelines recommend adjuvant systemic therapy in patients with early-stage NSCLC who have high-risk relapse factors after SBRT [41]. The definition of these high-risk relapse factors has yet to be explored. In our study, in the training cohort, patients were divided into high- and low-risk groups according to the optimal cutoff value (0.19). The Kaplan-Meier plots suggested that the DMFS of patients in the training cohort (*P* < 0.001, hazard ratio (HR) = 4.855, 95% CI = 2.537–9.293) and the external validation cohort (*P* < 0.001, HR = 13.021, 95% CI = 4.189–40.474) were significantly stratified by risk grouping. The risk of distant metastasis in the high-risk group was 13.021 times greater than that in the low-risk group, as indicated by the HR of the validation set. This demonstrated that a probability of distant metastasis in patients exceeding 0.19 significantly impacted DMFS and was considered a risk factor for distant metastasis, leading to a significantly increased risk. Figure 3 shows that the DMFS of the high-risk group decreased from 78.4% at 1 year to 44.1% at 5 years, while the DMFS of the low-risk group decreased from 96.8% at 1 year to 88.4% at 5 years. Patients at high risk of metastasis should receive closer follow-up and/or adjuvant systemic therapy.

Several retrospective studies have sought to identify patients who would benefit from systemic adjuvant therapy. Using the National Cancer Database (*n* = 7042), Grinnell confirmed that adjuvant chemotherapy could improve OS in patients with a tumor diameter ≥ 4 cm [42]. Ernani et al. also analyzed the National Cancer Database (*n* = 11,836) and obtained similar results [43]. However, distant metastasis can also occur in patients with a tumor diameter less than 4 cm. In these patients, adjuvant chemotherapy may improve their OS. The results of our study can provide a reference for the consideration of adjuvant chemotherapy in high-risk patients with a probability of distant metastasis greater than 0.19. In this study, when the tumor diameter was between 1 and 2 cm, the DMFS of low-risk patients was significantly better than that of high-risk patients (*P* < 0.001, HR = 8.158, 95% CI, 2.473–26.916). When the tumor diameter was between 2 and 3 cm, the difference in DMFS between the high- and low-risk groups was still significant (*P* < 0.001, HR = 5.242, 95% CI, 2.306–11.916). These results verify the reliability of the risk grouping according to the model. An ongoing trial (NCT03833154) has compared SBRT with SBRT plus immunotherapy in early stage unresected NSCLC. The results of this clinical trial will provide clear implications for the individualized treatment of early stage unresected NSCLC.

As a strength and innovation, the model presented in this study was validated in multiple institutions with a stable predictive value. A formula to calculate the probability of distant metastasis was proposed to assess the risk of distant metastasis. The adopted feature selection method was robust. In general, the most stable radiomics features were selected through 1000 bootstrap feature screenings, which was conducive to avoiding overly optimistic results. In addition, internal validation by the bootstrapping method could prevent overfitting, so that the results are representative [44]. Patients with or without pathological diagnoses were included in the study, which contributes to a more general prediction model that is potentially applicable to patients who cannot undergo biopsy because of contraindications. Our radiomics model showed equally good discrimination in patients with (AUC 0.816) and without (AUC 0.836) pathological diagnosis (Figure S5). The Kaplan-Meier plots suggested that the DMFS of patients with (*P* < 0.001, HR = 4.515, 95% CI, 2.301–8.861) and without (*P* < 0.001, HR = 9.546, 95% CI, 3.477–26.213) pathological diagnosis were both significantly stratified by risk grouping (Figure S6).

The following limitations of this study must be acknowledged. First, this study was retrospective, and the distribution of certain clinical characteristics of the patients was significantly different between the training and external validation sets. Nevertheless, the constructed prognostic model had good predictive value for distant metastasis in all sets. Second, we resampled the PET images as spacings of 2 × 2 × 2 pixels; however, their effects on the features remained unknown. Because there was no definitive conclusion on the optimal parameters for radiomics research, we set the parameter settings for extracting features in the supplementary material A1 [45]. More basic research on radiomics is needed to determine the optimal parameter settings to improve the generalizability and stability of the radiomics model. To achieve successful multi-institutional validation of radiomics, several challenges must be addressed. These challenges include data heterogeneity across different institutions, algorithm repeatability and reliability [46], data sharing and privacy protection, and biological validation. To effectively overcome these challenges, several strategies can be employed. These include data standardization, strict quality control and validation of algorithms, data desensitization, and correlation analysis of radiomics with other biological data, such as genomics, proteomics, or pathology data, which can be used for biological validation. Our research direction also aligns with these strategies, aiming to establish a model that can be widely applied to multi-institution prediction of distant metastasis in the future.

## Conclusion

The combination of PET and CT radiomic features improves the prediction of distant metastasis in patients with early-stage NSCLC treated with SBRT. The proposed radiomics model can be used for the prediction of distant metastasis and to guide the personalized treatment of patients with different prognoses.

### Electronic supplementary material

Below is the link to the electronic supplementary material.

**Supplementary Material 1:** Supplementary methods, tables and figures

## Data Availability

The datasets are available from the corresponding author on reasonable request.

## Abbreviations

AIC: Akaike information criterion
AUC: Area under the curve
BED: Biologically equivalent dose
CI: Confidence interval
CT: Computed tomography
DCA: Decision curve analysis
DICOM: Digital Imaging and Communications in Medicine
DMFS: Distant metastasis-free survival
ECOG: Eastern Cooperative Oncology Group
GLCM: Gray Level Cooccurrence Matrix
GLRLM: Gray Level Run Length Matrix
GLSZM: Gray Level Size Zone Matrix
HR: Hazard ratio
KSTM: Kernel-backed tensor machine
LASSO: Least absolute shrinkage and selection operator
LR: Logistic regression
MRI: Magnetic Resonance Imaging
NCCN: National Comprehensive Cancer Network
NGTDM: Neighboring gray tone difference matrix
NSCLC: Non-small cell lung cancer
PET/CT: Positron emission tomography/computed tomography
RMS: RootMeanSquared
ROC: Receiver operating characteristic curve
SBRT: Stereotactic body radiotherapy
SUVmax: Maximum Standardized Uptake Value
TKI: Tyrosine kinase inhibitor
^18^F-FDG: ^18^F-Fluorodeoxyglucose
