# Supplementary material for: A PET/CT radiomics model for predicting distant metastasis in early-stage non–small cell lung cancer patients treated with stereotactic body radiotherapy: a multicentric study
Source: Radiat Oncol. 2024 Jan 22;19:10. doi: 10.1186/s13014-024-02402-z (PMC10802016; doi:10.1186/s13014-024-02402-z)
Supplement: Supplementary file 1 — Supplementary Material 1: Supplementary methods, tables and figures [file 13014_2024_2402_MOESM1_ESM.docx]

**Supplementary Material**

[Supplementary Methods 2](#_Toc155081558)

[A1. Radiomics procedure 2](#_Toc155081559)

[A2. The formulas of the radiomics score 4](#_Toc155081562)

[Supplementary Tables 5](#_Toc155081563)

[Table S1. Information of datasets from different institutions. 5](#_Toc155081564)

[Table S2. Acquisition and reconstruction of PET/CT. 6](#_Toc155081565)

[Table S3. Elucidation of the radiomics features extracted. 7](#_Toc155081566)

[Table S4. The top fifteen frequent radiomics features. 7](#_Toc155081567)

[Table S5. The top three frequent radiomics signatures. 8](#_Toc155081568)

[Table S6. Subgroup analysis between the CT radiomics score and distant metastasis status. 9](#_Toc155081569)

[Supplementary Figures 10](#_Toc155081570)

[Figure S1. Screening pathways for patients. 10](#_Toc155081571)

[Figure S2. Radiomics feature frequency rank. 11](#_Toc155081572)

[Figure S3. Model comparisons with conventional clinical parameters. 12](#_Toc155081573)

[Figure S4. Decision curve analysis of the combined radiomics model. 13](#_Toc155081574)

[Figure S5. Performance of the combined radiomics signature in patients with and without pathological diagnosis. 13](#_Toc155081575)

[Figure S6. Kaplan-Meier analysis of patients with and without pathological diagnosis. 14](#_Toc155081576)

[Supplementary References 14](#_Toc155081577)

Supplementary Methods

A1. Radiomics procedure

**Tumor segmentation**

We use 3D-Slicer software (version 4.13.0) to segment tumor (1, 2). All lesions were delineated by a radiologist with 8 years of experience (Xiaofeng Wang) and modified by a senior radiologist with 16 years of experience (Yong Guan).

Radiomics features were extracted from DICOM CT and PET using O-RAW package (based on Pyradiomics v3.7) (3). These features comprised 17 intensity histogram features, 13 morphological (shape) features, and 73 textural features. No digital image filters were applied during pre-processing. Most of the hand-crafted features conformed to the Image Biomarker Standardization Initiative (IBSI) (4). Supplementary Table S3 shows all the radiomics features.

**Radiomics features extraction parameter settings**

*# CT:*

*imageType:*

*Original:*

*binWidth: 25*

*featureClass:*

*shape:*

*- VoxelVolume*

*- Elongation*

*- Flatness*

*- LeastAxisLength*

*- MajorAxisLength*

*- Maximum2DDiameterColumn*

*- Maximum2DDiameterRow*

*- Maximum2DDiameterSlice*

*- Maximum3DDiameter*

*- MeshVolume*

*- MinorAxisLength*

*- Sphericity*

*- SurfaceArea*

*- SurfaceVolumeRatio*

*firstorder: # Remove Total Energy, correlated to Energy (due to resampling enabled)*

*- 10Percentile*

*- 90Percentile*

*- Energy*

*- Entropy*

*- InterquartileRange*

*- Kurtosis*

*- Maximum*

*- Mean*

*- MeanAbsoluteDeviation*

*- Median*

*- Minimum*

*- Range*

*- RobustMeanAbsoluteDeviation*

*- RootMeanSquared*

*- Skewness*

*- Uniformity*

*- Variance*

*glcm: # Disable SumAverage by specifying all other GLCM features available*

*- 'Autocorrelation'*

*- 'JointAverage'*

*- 'ClusterProminence'*

*- 'ClusterShade'*

*- 'ClusterTendency'*

*- 'Contrast'*

*- 'Correlation'*

*- 'DifferenceAverage'*

*- 'DifferenceEntropy'*

*- 'DifferenceVariance'*

*- 'JointEnergy'*

*- 'JointEntropy'*

*- 'Imc1'*

*- 'Imc2'*

*- 'Idm'*

*- 'Idmn'*

*- 'Id'*

*- 'Idn'*

*- 'InverseVariance'*

*- 'MaximumProbability'*

*- 'SumEntropy'*

*- 'SumSquares'*

*glrlm:*

*glszm:*

*gldm:*

*ngtdm:*

*setting:*

*# Resampling:*

*resampledPixelSpacing: [2, 2, 2]*

*padDistance: 10 # Extra padding for large sigma valued LoG filtered images*

*# Resegmentation: remove outliers >3 std from the mean (affects all classes except shape)*

*resegmentRange: [-3, 3]*

*resegmentMode: sigma*

*# first order specific settings:*

*voxelArrayShift: 1000*

*label: 1*

*# PET:*

*imageType:*

*Original:*

*binWidth: 0.3*

*featureClass:*

*shape:*

*- VoxelVolume*

*- Elongation*

*- Flatness*

*- LeastAxisLength*

*- MajorAxisLength*

*- Maximum2DDiameterColumn*

*- Maximum2DDiameterRow*

*- Maximum2DDiameterSlice*

*- Maximum3DDiameter*

*- MeshVolume*

*- MinorAxisLength*

*- Sphericity*

*- SurfaceArea*

*- SurfaceVolumeRatio*

*firstorder: # Remove Total Energy, correlated to Energy (due to resampling enabled)*

*- 10Percentile*

*- 90Percentile*

*- Energy*

*- Entropy*

*- InterquartileRange*

*- Kurtosis*

*- Maximum*

*- Mean*

*- MeanAbsoluteDeviation*

*- Median*

*- Minimum*

*- Range*

*- RobustMeanAbsoluteDeviation*

*- RootMeanSquared*

*- Skewness*

*- Uniformity*

*- Variance*

*glcm: # Disable SumAverage by specifying all other GLCM features available*

*- 'Autocorrelation'*

*- 'JointAverage'*

*- 'ClusterProminence'*

*- 'ClusterShade'*

*- 'ClusterTendency'*

*- 'Contrast'*

*- 'Correlation'*

*- 'DifferenceAverage'*

*- 'DifferenceEntropy'*

*- 'DifferenceVariance'*

*- 'JointEnergy'*

*- 'JointEntropy'*

*- 'Imc1'*

*- 'Imc2'*

*- 'Idm'*

*- 'Idmn'*

*- 'Id'*

*- 'Idn'*

*- 'InverseVariance'*

*- 'MaximumProbability'*

*- 'SumEntropy'*

*- 'SumSquares'*

*glrlm:*

*glszm:*

*gldm:*

*ngtdm:*

*setting:*

*# Resampling:*

*resampledPixelSpacing: [2, 2, 2]*

*padDistance: 10*

*# Resegmentation: remove outliers >3 std from the mean (affects all classes except shape)*

*resegmentRange: [-3, 3]*

*resegmentMode: sigma*

*voxelArrayShift: 0*

*label: 1*

A2. The formulas of the radiomics score

*CT radiomics score=-2.77309*original_gldm_DependenceEntropy
+0.0000501557*original_gldm_LargeDependenceHighGrayLevelEmphasis
-353.988*original_glrlm_LowGrayLevelRunEmphasis
+18.6451*

*PET radiomics score =-0.955928*original_firstorder_RootMeanSquared
+11.6029*original_glcm_Idm
+1.63572*original_glcm_JointEntropy
+0.00131486*original_glcm_SumSquares
-15.7597*

Supplementary Tables

Table S1. Information of datasets from different institutions.

| **Institution** | **City** | **Start date** | **End date** | **Number of patients** |
| --- | --- | --- | --- | --- |
| Tianjin Medical University Cancer Institute and Hospital | Tianjin | July 2012 | October 2020 | 139 |
| Zhejiang Cancer Hospital | Hangzhou | April 2017 | September 2018 | 20 |
| Affiliated Tumor Hospital of Zhengzhou University | Zhengzhou | April 2016 | January 2021 | 27 |
| Fudan University Shanghai Cancer Center | Shanghai | May 2016 | November 2021 | 19 |
| Shandong Cancer Hospital and Institute | Jinan | July 2016 | August 2021 | 23 |

Table S2. Acquisition and reconstruction of PET/CT.

| **Acquisition**  **parameters** | **Discovery 690 – General Electric (TianJin)** | | **Discovery 710 – General Electric (ZheJiang)** | | **Discovery STE– General**  **Electric (ZhengZhou)** | | **Gemini TF Big Bore-**  **Philips (Shandong)** | | **Biograph 16-Siemens**  **(Shanghai)** | |
| --- | --- | --- | --- | --- | --- | --- | --- | --- | --- | --- |
|  | PET | CT | PET | CT | PET | CT | **PET** | **CT** | **PET** | **CT** |
| **^18^F-FDG activity**  **(MBq)** | 370–740 | - | 370–740 | - | 370–740 | - | 370–740 | - | 370–740 | - |
| **Min/bed position** | 2 | - | 2 | - | 2 | - | 2 | - | 2 | - |
| **Crystal** | LYSO | - | LYSO | - | LYSO | - | LYSO | - | LYSO | - |
| **Reconstruction** | Iterative,TOF  Sharp IR | - | Iterative,TOF  Sharp IR | - | Iterative,TOF  Sharp IR | - | Iterative,TOF  Sharp IR | - | Iterative,TOF  Sharp IR | - |
| **Matrix (pixels)** | 192×192 | 512×512 | 192×192 | 512×512 | 128×128 | 512×512 | 144×144 | 512×512 | 168×168 | 512×512 |
| **Resolution (mm)** | 3.65×3.65 | 1.37×1.37 | 3.65×3.65 | 0.98×0.98 | 5.5×5.5 | 0.98×0.98 | 4×4 | 1.17×1.17 | 4.1×4.1 | 1.37×1.37 |
| **Slice thickness (mm)** | 3.27 | 3.75 | 3.27 | 3.75 | 3.27 | 3.75 | 5.00 | 5.00 | 5.00 | 5.00 |
| **Slices** | - |  | - |  | - |  | - |  | - |  |
| **Voltage (kV)** | - | 120 | - | 140 | - | 120 | - | 120 | - | 120 |
| **Tube current (mA)** | - | 194 | - | 171 | - | 207 | - | 206 | - | 143 |
| **Reconstruction**  **Method** | VPFXS | - | VPFXS | - | 3D IR | - | VPFXS | - | VPFXS | - |
| **Correction Applied** | Decy,attn,scat,dtim,ransng,dcal,slsens,norm |  | Decy,attn,scat,dtim,ransng,dcal,slsens,norm |  | Decy,attn,scat,dtim,ran,dcal,slsens,norm |  | Decy,radl,attn,scat,dtim,ran,norm |  | Norm,ntim,attn,scat,radl,decy |  |

Table S3. Elucidation of the radiomics features extracted.

| **Group** | **Subgroup** | **Radiomics Features** |
| --- | --- | --- |
| First-order statistics features |  | 10Percentile, 90Percentile, Energy, Entropy, InterquartileRange, Kurtosis, Maximum, Mean, MeanAbsoluteDeviation, Median, Minimum, RobustMeanAbsoluteDeviation, Range, RootMeanSquared, Skewness, Uniformity, Variance |
| Shape-based features |  | Elongation, Flatness, LeastAxisLength, MajorAxisLength, Maximum2DDiameterColumn, Maximum2DDiameterRow, Maximum2DDiameterSlice, Maximum3DDiameter, MeshVolume, MinorAxisLength, Sphericity, SurfaceArea, SurfaceVolumeRatio |
| Statistics-based textural features | GLCM | Autocorrelation, ClusterProminence, ClusterShade, ClusterTendency, Contrast, Correlation, DifferenceAverage, DifferenceEntropy, DifferenceVariance, Id, Idm, Idmn, Idn, Imc1, Imc2, InverseVariance, JointAverage, JointEnergy, JointEntropy, MaximumProbability, SumEntropy, SumSquares |
|  | GLRLM | GrayLevelNonUniformity, GrayLevelNonUniformityNormalized, GrayLevelVariance, HighGrayLevelRunEmphasis, LongRunEmphasis, LongRunHighGrayLevelEmphasis, LongRunLowGrayLevelEmphasis, LowGrayLevelRunEmphasis, RunEntropy, RunLengthNonUniformity, RunLengthNonUniformityNormalized, RunPercentage, RunVariance, ShortRunEmphasis, ShortRunHighGrayLevelEmphasis, ShortRunLowGrayLevelEmphasis |
|  | GLSZM | GrayLevelNonUniformity, GrayLevelNonUniformityNormalized, GrayLevelVariance, HighGrayLevelZoneEmphasis, LargeAreaEmphasis, LargeAreaHighGrayLevelEmphasis, LargeAreaLowGrayLevelEmphasis, LowGrayLevelZoneEmphasis, SizeZoneNonUniformity, SizeZoneNonUniformityNormalized, SmallAreaEmphasis, SmallAreaHighGrayLevelEmphasis, SmallAreaLowGrayLevelEmphasis, ZoneEntropy, ZonePercentage, ZoneVariance |
|  | NGTDM | Busyness, Coarseness, Complexity, Contrast, Strength |
|  | GLDM | DependenceEntropy, DependenceNonUniformity, DependenceNonUniformityNormalized, DependenceVariance, GrayLevelNonUniformity, GrayLevelVariance, HighGrayLevelEmphasis, LargeDependenceEmphasis, LargeDependenceHighGrayLevelEmphasis, LargeDependenceLowGrayLevelEmphasis, LowGrayLevelEmphasis, SmallDependenceEmphasis, SmallDependenceHighGrayLevelEmphasis, SmallDependenceLowGrayLevelEmphasis |

GLSZM: Gray Level Size Zone Matrix; GLRLM: Gray Level Run Length Matrix; GLDM: Gray Level Dependence Matrix; GLCM: Gray Level Cooccurrence Matrix; NGTDM, neighboring gray tone difference matrix.

Table S4. The top fifteen frequent radiomics features.

**Table S4A.** The top fifteen frequent CT radiomics features.

| **No.** | **CT radiomics features** | **Frequency** |
| --- | --- | --- |
| 1. | original_glrlm_LowGrayLevelRunEmphasis | 804 |
| 2. | original_gldm_LargeDependenceHighGrayLevelEmphasis | 735 |
| 3. | original_gldm_DependenceEntropy | 603 |
| 4. | original_shape_Maximum2DDiameterRow | 575 |
| 5. | original_glcm_JointEnergy | 529 |
| 6. | original_glcm_ClusterProminence | 491 |
| 7. | original_glszm_SmallAreaLowGrayLevelEmphasis | 425 |
| 8. | original_firstorder_Kurtosis | 410 |
| 9. | original_glrlm_LongRunHighGrayLevelEmphasis | 408 |
| 10. | original_glszm_ZoneVariance | 394 |
| 11. | original_ngtdm_Strength | 351 |
| 12. | original_glszm_SizeZoneNonUniformity | 295 |
| 13. | original_shape_SurfaceVolumeRatio | 287 |
| 14. | original_shape_MinorAxisLength | 270 |
| 15. | original_firstorder_Median | 268 |

**Table S4B.** The top fifteen frequent PET radiomics features.

| **No.** | **PET radiomics features** | **Frequency** |
| --- | --- | --- |
| 1. | original_shape_SurfaceVolumeRatio | 897 |
| 2. | original_shape_Elongation | 629 |
| 3. | original_firstorder_Median | 534 |
| 4. | original_glszm_LargeAreaEmphasis | 449 |
| 5. | original_shape_MinorAxisLength | 437 |
| 6. | original_gldm_DependenceNonUniformityNormalized | 424 |
| 7. | original_shape_LeastAxisLength | 386 |
| 8. | original_ngtdm_Strength | 378 |
| 9. | original_glcm_SumSquares | 313 |
| 10. | original_firstorder_InterquartileRange | 244 |
| 11. | original_gldm_LargeDependenceHighGrayLevelEmphasis | 240 |
| 12. | original_glcm_JointEntropy | 138 |
| 13. | original_gldm_LargeDependenceEmphasis | 117 |
| 14. | original_gldm_SmallDependenceHighGrayLevelEmphasis | 117 |
| 15. | original_glszm_SmallAreaHighGrayLevelEmphasis | 98 |

Table S5. The top three frequent radiomics signatures.

**Table S5A.** The top three frequent CT radiomics signatures.

| **No.** | **CT signatures** | **Frequency** |
| --- | --- | --- |
| 1. | original_gldm_DependenceEntropy  original_gldm_LargeDependenceHighGrayLevelEmphasis  original_glrlm_LowGrayLevelRunEmphasis | 222 |
| 2. | original_gldm_DependenceEntropy  original_glrlm_LongRunHighGrayLevelEmphasis  original_glrlm_LowGrayLevelRunEmphasis | 66 |
| 3. | original_firstorder_Kurtosis  original_glcm_JointEnergy  original_glrlm_LowGrayLevelRunEmphasis | 49 |

**Table S5B.** The top three frequent PET radiomics signatures.

| **No.** | **PET signatures** | **Frequency** |
| --- | --- | --- |
| 1. | original_firstorder_RootMeanSquared  original_glcm_Idm  original_glcm_JointEntropy  original_glcm_SumSquares | 74 |
| 2. | original_glcm_Idm  original_glcm_JointEntropy | 65 |
| 3. | original_glcm_Id  original_glcm_SumSquares  original_gldm_DependenceNonUniformityNormalized | 63 |

Table S6. Subgroup analysis between the CT radiomics score and distant metastasis status.

| **Variables** |  | **CT radiomics score** | |  |
| --- | --- | --- | --- | --- |
|  | **Subgroups** | **DM^+^** | **DM^-^** | ***P*-value** |
| Age (years)^a^ | >73 | -0.246(-0.965 to 0.796) | -1.905(-3.243 to-1.050) | <0.001 |
|  | ≤73 | -0.617(-1.397 to 0.933) | -2.277(-3.832 to -0.977) | <0.001 |
| Sex | male | -0.617(-1.410 to 0.972) | -1.894(-3.130 to -0.814) | 0.001 |
|  | female | -0.504(-1.368 to 0.458) | -2.461(-4.178 to -1.369) | <0.001 |
| smoke | yes | -0.381(-0.981 to 0.916) | -1.865(-2.952 to -0.666) | <0.001 |
|  | no | -0.505(-0.380 to 0.594) | -2.303(-3.879 to -1.322) | <0.001 |
| ECOG | 0 | -0.617(-1.424 to 0.382) | -2.057(-3.534 to -1.276) | <0.001 |
|  | 1 | -0.186(-0.946 to 0.992) | -1.901(-3.261 to -0.704) | <0.001 |
| Histology | yes | -0.453(-1.247 to 0.917) | -1.689(-2.807 to -0.840) | <0.001 |
|  | no | -0.404(-1.380 to 0.618) | -2.309(-3.825 to -1.339) | <0.001 |
| Tumor size | >2.2 | -0.112(-0.800 to 1.364) | -1.559(-2.339 to -0.524) | <0.001 |
|  | ≤2.2 | -0.961(-1.432 to 0.160) | -2.646(-4.629 to -1.501) | <0.001 |

*^a^* The cutoff values of these continuous variables defining the subgroups were chosen using the median in all patients. DM, distant metastasis; ECOG, Eastern Cooperative Oncology Group.

Supplementary Figures

Figure S1. Screening pathways for patients.

**
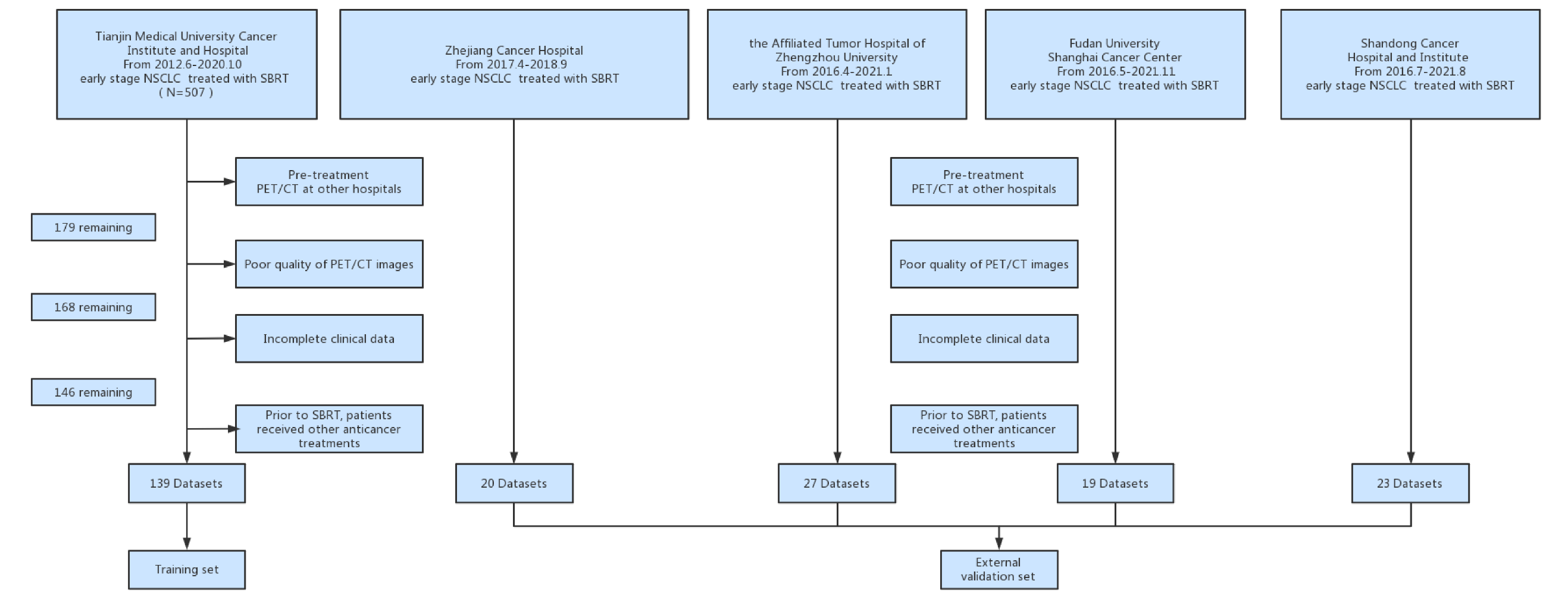
**

PET/CT, positron emission tomography/computed tomography.

**Figure S2.** Radiomics feature frequency rank.


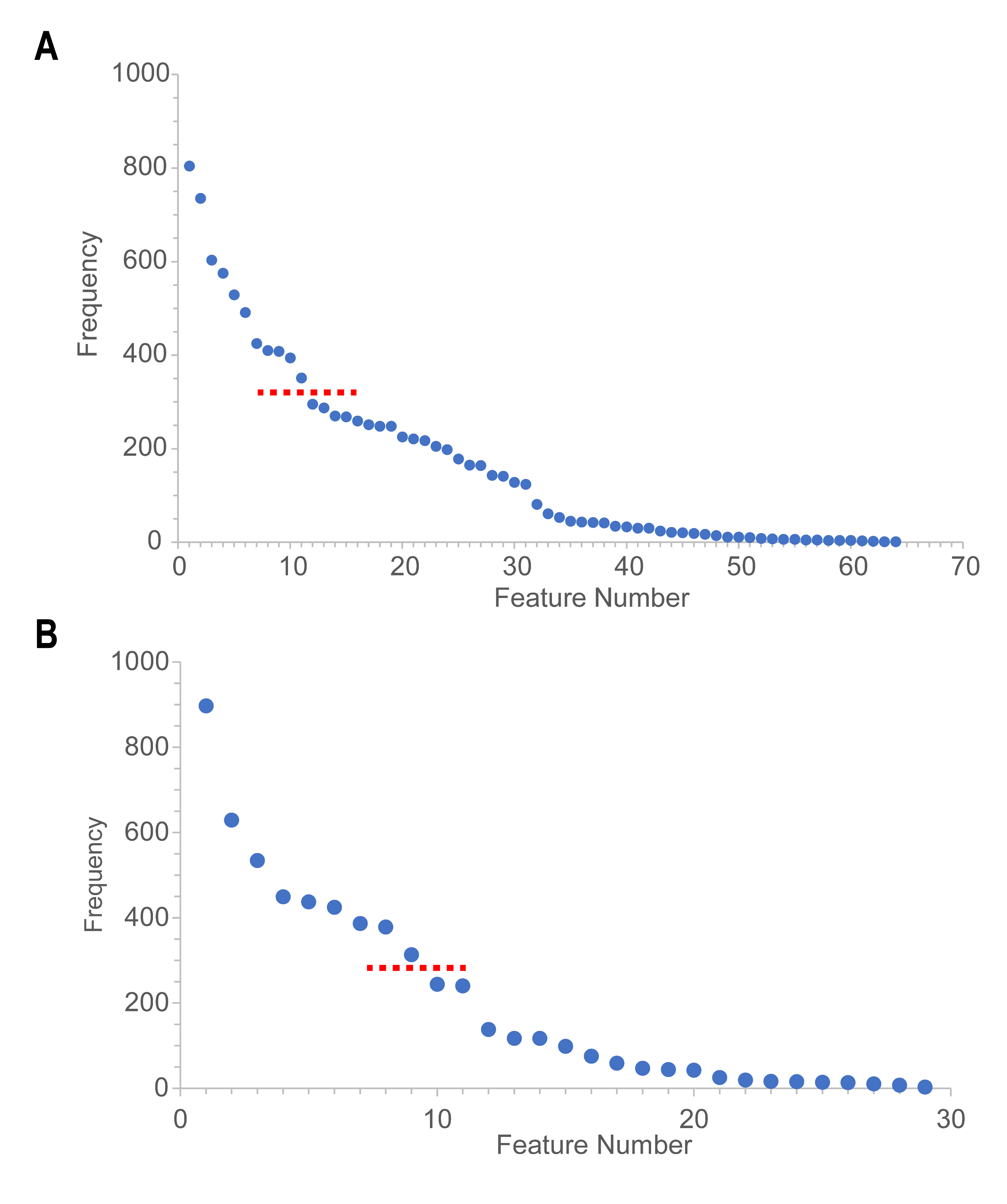


The radiomics features were sorted according to the number of frequencies, and the cutoff points were determined based on the frequency breakpoints shown in the graphs. **(A)** CT radiomics features. **(B)** PET radiomics features. CT, computed tomography. PET, positron emission tomography.

Figure S3. Model comparisons with conventional clinical parameters.

**
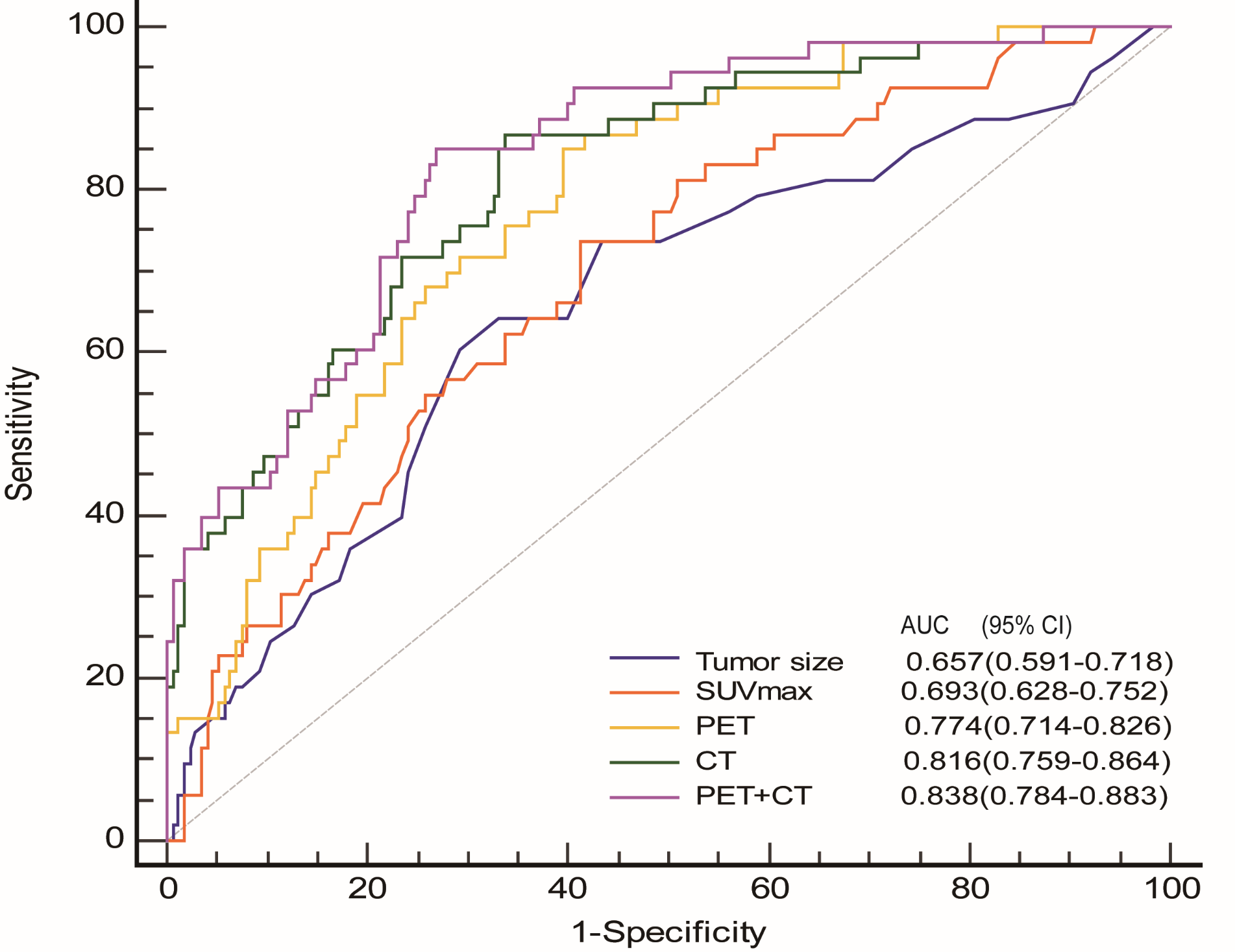
**

ROC curve analyses were used to compare the discriminatory efficacy of the radiomics model with those of the tumor size and SUVmax. ROC, receiver operator characteristic curve. AUC, area under the receiver operator characteristic curve. CI, confidence interval. CT, computed tomography. PET, positron emission tomography.

Figure S4. Decision curve analysis of the combined radiomics model.


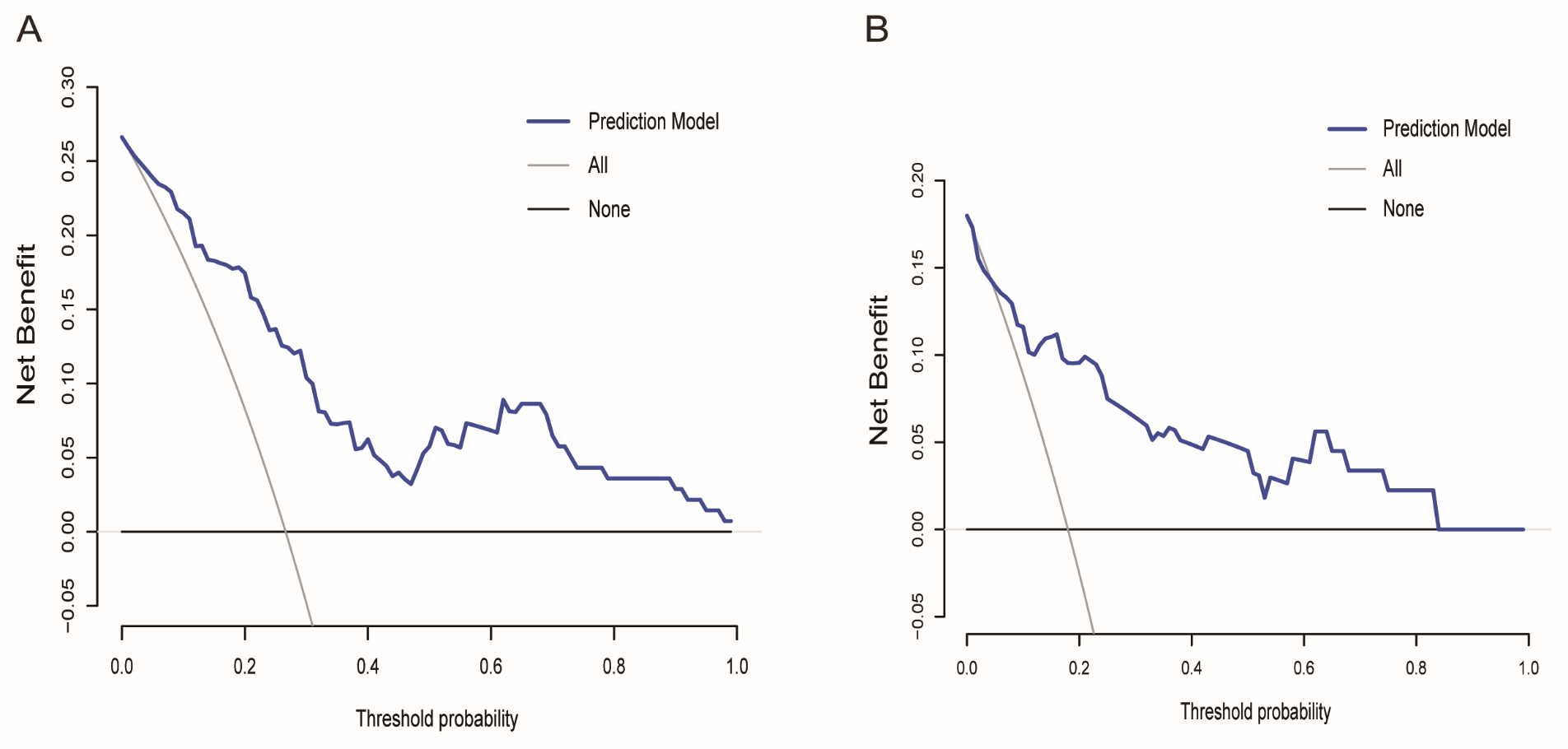


**(A)** The decision curve for the combined radiomics model in the training set with a probability of distant metastasis ranges from 0% to 100.0%. **(B)** The decision curve for the combined radiomics model in the external validation set with a probability of distant metastasis ranges from 0% to 82.0%.

Figure S5. Performance of the combined radiomics signature in patients with and without pathological diagnosis.

**
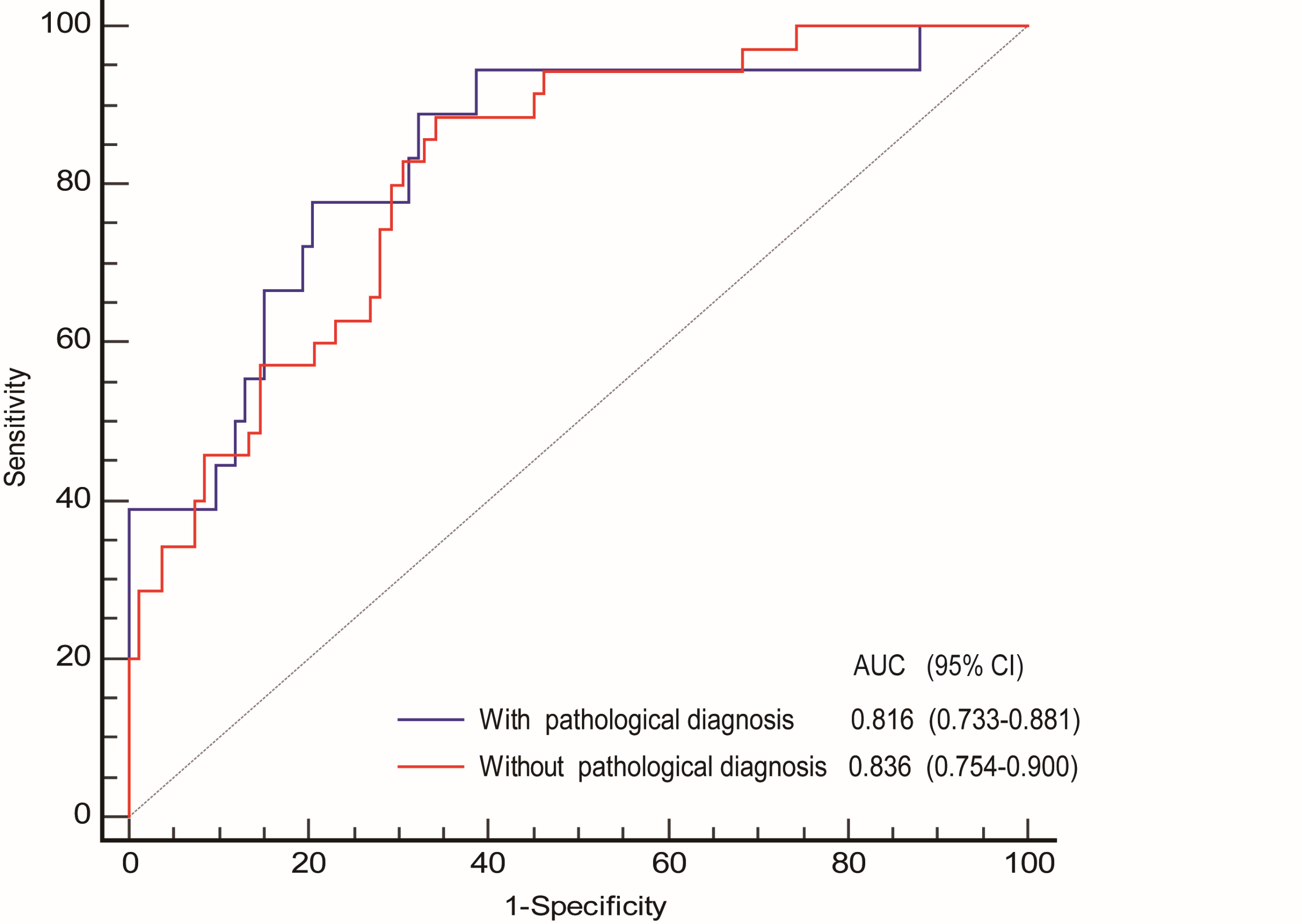
**

Performance of the combined radiomics signature in patients with and without pathological diagnosis.

Figure S6. Kaplan-Meier analysis of patients with and without pathological diagnosis.


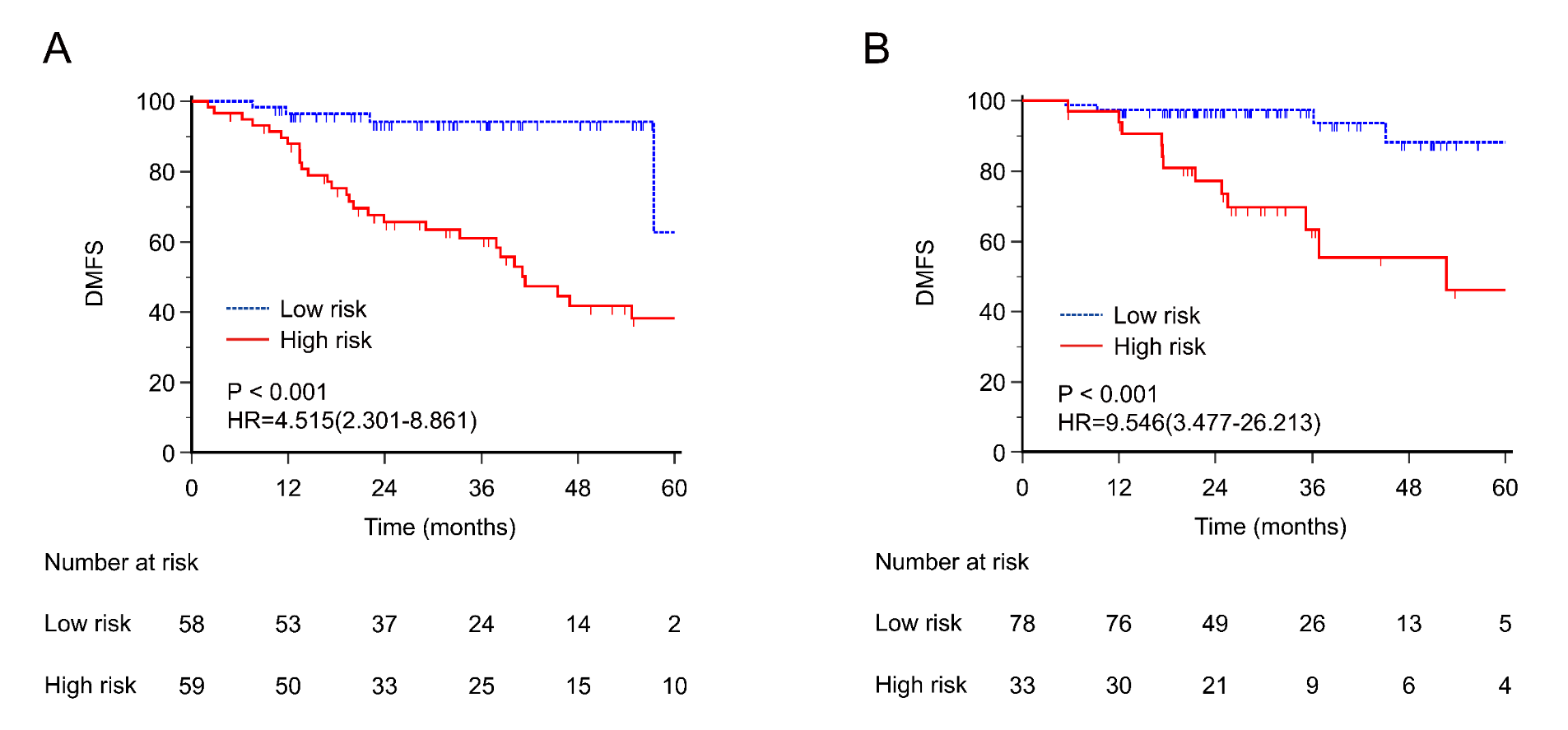

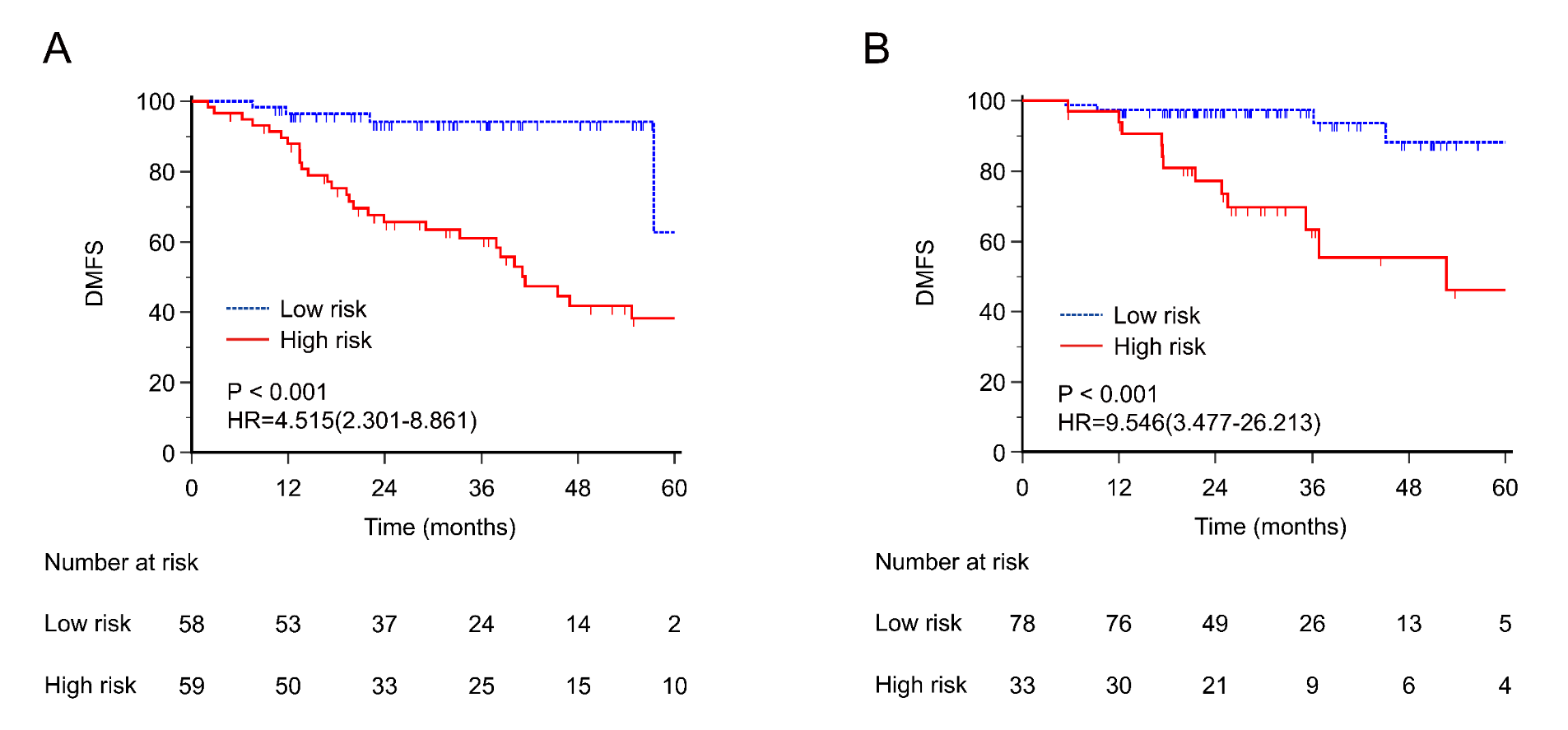


Kaplan-Meier analysis of distant metastasis free survival (DMFS) of patients with and without pathology, the population was divided into two risk groups according to predicted probability. **(A)** Patients with pathological diagnosis. **(B)** Patients without pathological diagnosis.

Supplementary References

1. Owens CA, Peterson CB, Tang C, Koay EJ, Yu W, Mackin DS, et al. Lung tumor segmentation methods: Impact on the uncertainty of radiomics features for non-small cell lung cancer. PLoS One. 2018;13(10):e0205003.

2. Jiménez Londoño GA, Pérez-Beteta J, Bosque JJ, Honguero-Martinez AF, García Vicente AM. Segmentation in Non-Small Cell Lung Tumors Using 18F-FDG PET/CT: Tips and Rules. Clin Nucl Med. 2020;45(11):e477-e82.

3. Liu Z, Wang S, Dong D, Wei J, Fang C, Zhou X, et al. The Applications of Radiomics in Precision Diagnosis and Treatment of Oncology: Opportunities and Challenges. Theranostics. 2019;9(5):1303-22.

4. Zwanenburg A, Vallières M, Abdalah MA, Aerts H, Andrearczyk V, Apte A, et al. The Image Biomarker Standardization Initiative: Standardized Quantitative Radiomics for High-Throughput Image-based Phenotyping. Radiology. 2020;295(2):328-38.
